# Examining the Connection Between Vascular Function and Cognitive Performance

**DOI:** 10.1002/alz70855_107028

**Published:** 2025-12-24

**Authors:** Enid Swatson, Whitney Wharton, Danielle D Verble, Chloe Park, Jordan Watson, Brian Kim, Bruno L. Hammerschlag, Hanfeng Huang, Brittany Butts

**Affiliations:** ^1^ Emory University, Atlanta, GA, USA; ^2^ Massachusetts General Hospital, Harvard Medical School, Boston, MA, USA; ^3^ Georgetown University School of Medicine, Washington, DC, USA

## Abstract

**Background:**

Cardiovascular disease remains a leading cause of morbidity and mortality among aging adults in the United States. Hypertension, high cholesterol, and modifiable behaviors including diet and inactivity contribute to cardiovascular dysfunction, and are independent risk factors for Alzheimer's disease (AD). Chronic hypertension accelerates arterial stiffness, reducing elasticity and impairing cerebral blood flow. Extended exposure to these conditions is linked to cognitive decline, especially in executive function and memory. The purpose of this study is to examine the relationship between vascular function and multiple cognitive domains in a racially diverse, cognitively normal cohort at risk for AD.

**Method:**

Participants are enrolled in either the E2 (currently enrolling) or the ASCEND study (PI Wharton). Both longitudinal cohorts include middle aged (45‐65yrs) cognitively normal individuals, most of who have an AD parental history. A one‐hour, in‐person cognitive battery examined domains including verbal fluency, visuospatial ability, and executive function. Pulse wave velocity (PWV), a measure of arterial stiffness, and aortic blood pressures were collected at the same visit. Linear regression analyses controlling for age and gender were performed.

**Result:**

Participants (*N* = 243) were mostly women (70%) college educated (84%), 47% Black /African American (B/AA), and average 61 years. PWV was negatively associated with FAS (β=‐0.388; *p* = .011) and CERAD (β=‐0.350; *p* = .02) among NHW, with no associations among B/AA individuals. Systolic blood pressure was negatively associated with digit span among NHWs (β = ‐0.268; *p* = 0.010) and B/AAs (β = ‐0.222; *p* = 0.0035). Diastolic blood pressure was negatively associated with MOCA (β = ‐0.209, *p* = 0.003) and digit span (β=0.240; *p* = .04) among NHW only. Pulse pressure was positively associated with Trails B time (β=0.435, *p* <.001) among NHW only.

**Conclusion:**

Higher blood pressure and arterial stiffness were associated with worse verbal fluency, working memory, visuospatial, and executive function. Findings support, and add to prior work, such that vascular and cognitive assessments may differ by race, and a one‐size‐fits‐all approach is not appropriate in delineating disease mechanisms and pathways. Future research should include diverse cohorts, most at risk for vascular disease and cognitive decline, to understand mechanisms, and ensure that diagnostics, treatments, and prevention strategies are appropriate and advantageous to all populations.

